# Coexisting Disadvantages in later Life: Demographic and Socio-Economic Inequalities

**DOI:** 10.1007/s12062-016-9158-y

**Published:** 2016-09-02

**Authors:** Josephine Heap, Stefan Fors, Carin Lennartsson

**Affiliations:** 1 Department of Social Work, Stockholm University, 106 91 Stockholm, Sweden; 2 Aging Research Center (ARC), Karolinska Institutet and Stockholm University, Gävlegatan 16, 113 30 Stockholm, Sweden

**Keywords:** Oldest old, Living conditions, Welfare, Inequality, Health

## Abstract

In this study, we aimed to identify which of certain demographic and socio-economic groups in the oldest part of the population that have an increased probability of experiencing simultaneous disadvantages in different life domains - here termed coexisting disadvantages. To do so, we compared analyses of coexisting disadvantages, measured as two or more simultaneous disadvantages, with analyses of single disadvantages and specific combinations of disadvantages. Indicators of physical health problems, ADL limitations, psychological health problems, limited financial resources, and limited social resources were included. We used nationally representative data from 2011 on people aged 76 and older in Sweden (*n* = 765). Results showed that coexisting disadvantages were associated with specific demographic and socio-economic groups, particularly certain marital status groups. Moreover, the differences between the demographic and socio-economic groups were only found for those who reported coexisting disadvantages, and not for those who reported only one disadvantage, which suggests that demographic and social factors become more important as disadvantages compound. Further, we analysed pairwise combinations of disadvantages. We found that different combinations of disadvantages tended to be associated with different groups, information useful from a social planning perspective since different combinations of disadvantages may imply different needs for help and support.

## Introduction

People of advanced old age generally have an increased probability of experiencing simultaneous disadvantages in more than one life domain (Barnes et al. 2006; Halleröd 2009; Tsakloglou and Papadopoulos 2002) – here termed coexisting disadvantages. In this study, we aimed to explore to what extent coexisting disadvantages are unequally distributed between demographic and socio-economic groups in the oldest part of the population. By identifying particularly vulnerable groups, the results could potentially serve as a basis for efficient social policy planning.

Old age has been described as a time of cumulative loss (Dean 2009) because older people are vulnerable to disadvantages in several life domains. For decades, older people were more exposed to poverty than people of working age. Although age-related patterns of poverty have changed in several western societies over the past several decades (Gustafsson et al. 2009; Moffatt and Scambler 2008), older people remain reliant on transfers (e.g. pensions) for their financial well-being to a greater extent than people of working age. This makes older people more vulnerable in this life domain, since they have fewer opportunities to influence their financial situation than people who are active in the labour force.

Older age is also associated with health problems and functional limitations. In addition to being an obstacle in itself, poor health may limit the capacity to maintain resources in other life domains, such as social contacts and activities. Several studies have found that disadvantages tend to appear simultaneously in different life domains (e.g. Erikson and Tåhlin 1987; Ferraro et al. 2009; Halleröd and Larsson 2008; Whelan and Maître 2008). Coexisting disadvantages have been defined as ‘society’s most pressing welfare problem’ (Esping-Andersen 2000). Among people of working age, the probability of experiencing coexisting disadvantages is associated with certain demographic and socio-economic characteristics, such as social class and marital status or household composition. Studying such differences is a way to analyse broad patterns of social inequality (Fritzell and Lundberg 2000). However, few studies have analysed how coexisting disadvantages are related to demographic and socio-economic characteristics among the oldest old people. It is plausible that patterns of social inequality similar to those identified among people of working age also emerge among older people. However, the older population is in several ways a distinct group that is shaped by, amongst other factors, cumulative inequality and selective mortality.

Cumulative inequality theory (Ferraro et al. 2009) postulates that social inequalities emerges early in life and accumulate over the life course. Drawing on the concept cumulative disadvantage (Dannefer 1987, 2003; O’Rand 1996), one of the tenets of cumulative inequality theory is that a disadvantage in one life domain increases the probability of exposure to other disadvantages within the same life domain, and may increase the exposure to risk in other life domains. Moreover, advantage increases the probability of exposure to further advantages. Cumulative inequality theory also recognises that cumulative inequality may lead to selective mortality. If risks accumulate to influence health, the most disadvantaged individuals may face premature mortality. Consequently, the older population has been described as an elite (Ferraro et al. 2009), because it consists of people who have survived into old age.

### Characteristics of Coexisting Disadvantages in Old Age

Among people of working age, limited financial resources are associated with a range of disadvantages and thus often constitute the core of coexisting disadvantages among people of working age (Fritzell and Lundberg 2000; Halleröd and Bask 2008; Korpi et al. 2007). Among older people, studies have found that when several different disadvantages are experienced at once, one of them is often physical health problems (Heap and Fors 2015; Heap et al. 2013; Whelan and Maître 2008). Moreover, among older adults, physical health problems are associated with several other kinds of disadvantages, including indicators of psychological health problems (Alexopoulos 2005; Blazer 2003; Wolitzky-Taylor et al. 2010), low financial resources (Grundy and Sloggett 2003; Kahn and Pearlin 2006), and limited social contacts and social support (Wong and Waite 2015).

### Demographic and Social Patterning of Coexisting Disadvantages

Studies tend to suggest that younger age is associated with an increased probability of experiencing coexisting disadvantages across different life domains (Halleröd and Larsson 2008; Korpi et al. 2007; Muffels and Fouarge 2004; Whelan and Maître 2008), but these studies often exclude the oldest old people or refrain from distinguishing between young-old and old-old people. A recent Swedish study found that people of advanced old age were more exposed to coexisting disadvantages than people between 18 and 75 (Heap et al. 2013). Moreover, several studies of older people have found that old-old people are more likely than young-old people to experience coexisting disadvantages – a pattern that emerged despite certain dissimilarities in the indicators of disadvantage that were included in the studies (Barnes et al. 2006; Halleröd 2009; Tsakloglou and Papadopoulos 2002). In contrast, however, a recent study focusing on psychosocial distress among older people found that those around retirement age (55 to 65) had a higher probability of experiencing multiple problems than people of advanced old age (81 to 99) (Bask 2015). To summarise, although indicators of disadvantages vary somewhat between studies, and this variance may affect patterns of age inequalities, the preponderance of study results suggests that in the older segment of the population, older age generally implies an increased probability of coexisting disadvantages.

Patterns of differences between women and men are somewhat unclear. Results differ, even between studies from the same country. Some Swedish studies of people of working age have found no gender differences in the probability of experiencing coexisting disadvantages (Ferrarini et al. 2010; Korpi et al. 2007); others, a higher probability in men (Bask 2010); and still others, a higher probability in women (Halleröd and Larsson 2008). Studies show that among older people, being female increases the likelihood of experiencing multiple psychosocial problems (Bask 2015) and coexisting disadvantages (Halleröd 2009). Another study found similar patterns, but only up to age 85. Among people of advanced old age, differences in coexisting disadvantages between women and men seemed to have diminished (Heap et al. 2013).

Patterns of coexisting disadvantages by social class seem to be more consistent. Several studies suggest that among both older and younger people in Sweden, manual workers have a higher probability of experiencing coexisting disadvantages than non-manual workers (Fritzell and Lundberg 2000; Halleröd 2009; Halleröd and Larsson 2008; Heap et al. 2013; Korpi et al. 2007). Marital status and household composition also seem to be associated with coexisting disadvantages across the whole adult age span. Studies from several different countries have shown that people in single-headed households; for example, single people, divorced people, lone parents, and widowed people, have a higher probability of experiencing coexisting disadvantages than married or cohabiting people (Barnes et al. 2006; Bask 2010; Halleröd 2009; Heap et al. 2013; Muffels and Fouarge 2004; Whelan and Maître 2007).

Several studies examine the simultaneous occurrence of two or more disadvantages but do not consider specific combinations of disadvantages. Researchers have noted that analysing coexisting disadvantages only as a specific number of disadvantages may blur more detailed patterns of stratification. When studying social exclusion (defined as multidimensional resource weaknesses), Barnes et al. (2006) noted that ‘there are a number of characteristics of older people that increase their odds of exclusion. .. these characteristics can vary according to the dimension of exclusion in question’ (Barnes et al. 2006: p. 36). Results from various research fields support the argument that the multiple indicators that can be included in the concept of coexisting disadvantages may be associated with different socio-economic and demographic characteristics. For example, among older people, women consistently experience more psychological health problems (Halleröd 2009; Wolitzky-Taylor et al. 2010; Zunzunegui et al. 2007) and limited financial resources (Arber and Ginn 2004; Barnes et al. 2006; Halleröd 2009; Lennartsson and Lundberg 2007) than men. On the other hand, it is not clear whether patterns of social contact differ between women and men (Ajrouch et al. 2005; Antonucci et al. 2002; Scheepers et al. 2002). Moreover, social class has been associated with physical health among older people (Enroth et al. 2013; Fors et al. 2008), but it is not clear whether social class is associated with psychological health. Consequently, analysing specific combinations of disadvantages may shed new light on the relationship between coexisting disadvantages and different demographic and socio-economic groups.

## Aim

The main aim of this study was to analyse the associations between coexisting disadvantages and different demographic and social groups of older people, and whether any such associations differ for different combinations of disadvantages. More specifically, we investigated possible differences in the likelihood of coexisting disadvantages by age, gender, social class, and marital status. We compared analyses of coexisting disadvantages, measured as two or more simultaneous disadvantages, with analyses of single disadvantages and specific combinations of disadvantages. Indicators of physical health problems, psychological health problems, limited financial resources, and limited social resources were included. These are all central indicators both in Scandinavian welfare research and in other research fields concerned with individuals’ welfare or well-being (Allardt 1993; Johansson 1970; Stiglitz et al. 2009).

## Methods

### Data

Data from the 2011 Swedish Panel Study of Living Conditions of the Oldest Old (SWEOLD) were used. The SWEOLD 2011 study consisted of a nationally representative sample of people aged 76 and older; respondents ranged in age between 76 and 101 years (*n* = 931). In 2011, SWEOLD oversampled people aged 85 and older, and we adjusted for this oversampling in the analyses. Both people who live in the community and people who live in institutions are included in SWEOLD. The 2011 response rate was 86.2 %. The participants represented the population well, for example in gender and age distribution and in the proportion who lived in an institutional care setting (Lennartsson et al. 2014). Most of the interviews were conducted face to face (57.8 %). When this was not possible, telephone interviews or postal questionnaires were used. If a respondent was unable to answer the questions, mainly due to frailty or cognitive impairment, proxy interviews were carried out with a relative or other close person (20.1 %). The SWEOLD study includes a wide thematic range of questions that provide comprehensive information about the living conditions of the oldest old people in Sweden. For a more detailed description of the SWEOLD survey, Lennartsson et al. (2014).

### Variables

#### Disadvantages

*Limited social resources* were measured with two indicators: one indicator of social isolation and one indicator of social support. The indicator of social isolation was based on four questions about social visits. Respondents were asked if they usually visited relatives, had relatives over for visits, visited friends, or had friends over for visits. Response alternatives were ‘No′, ‘Yes, sometimes’, and ‘Yes, often’. Those who responded ‘No′ to all four questions were considered socially isolated.

The indicator of social support included questions on instrumental support, emotional support, and having company. Instrumental support was measured with the question ‘Do you have a relative or close friend who is willing to help if you are ill?’ Emotional support was measured with the question ‘Do you have a relative or close friend who can help you if you need someone to talk to about personal problems?’ Having company was measured with the question ‘Do you have a relative or close friend who is willing to help if you want company?’ Response alternatives to all three questions were ‘Yes’ and ‘No′. Those who answered ‘No′ to at least one question were considered to have limited social support.

In the overall variable of social resources, those classified as socially isolated, as having limited social support, or both were considered to have limited social resources.

*Limited financial resources* were operationalised as a cash margin. To determine the presence or absence of such a financial buffer, respondents were asked if they could come up with 14,000 SEK (approx. £1180) in a week’s time. Those who were not able to come up with the money in a week’s time by drawing on their own resources – through a withdrawal from their own bank account or selling stocks and shares – were classified as having limited financial resources.

*Psychological health problems* were measured with three self-reported questions. Respondents were asked if they had experienced a number of ailments and diseases during the past 12 months. These included nervous problems (nervousness, anxiety, anguish), depression/deep sadness, and mental illness. Response alternatives were ‘Yes, severe’, ‘Yes, slight’, and ‘No′. Those who reported at least one of the following alternatives were considered to have psychological health problems: severe nervous problems, severe depression, and mild or severe mental illness.^1^

*Limitations in activities of daily living (ADL limitations)* were measured with five questions on the respondent’s ability to perform various tasks without any help from another person. The tasks were eating, toilet visits, dressing and undressing, getting into and out of bed, and hair washing. Those who were unable to perform at least one of the tasks independently were considered to have ADL limitations.

*Physical health problems* were measured with an indicator of circulatory problems and several items for other symptoms and diseases. The indicator of circulatory problems and the other symptoms and diseases were all drawn from the question ‘Have you had any of the following illnesses or ailments during the past 12 months?’ This question was followed by a list of specific health problems. Response alternatives were ‘Yes, severe’, ‘Yes, slight’, and ‘No′.

Circulatory problems were measured on the basis of the responses to questions about chest pains, heart problems, high blood pressure, dizziness, and coronary heart disease. Respondents were considered to have circulatory problems if they reported one severe or three slight problems with chest pains, heart problems, high blood pressure and/or dizziness, or if they reported slight or severe coronary heart disease.

Single items from the list of physical health problems were also used to assess the presence of the following symptoms and diseases: severe problems with breathlessness, severe problems with diabetes, severe problems with rheumatism, and severe problems with vision/eye disease that could not be substantially improved with glasses. Moreover, experience of mild or severe cancer, experience of mild or severe stroke, and presence of mild or severe Parkinson’s disease were also assessed using responses to items on the list.

In the overall variable of physical health problems, people were classified as having physical health problems if they reported circulatory problems or any of the symptoms and diseases measured with single items.^2^

#### Demographic and Socio-Economic Variables

*Age* was included as a continuous variable, and *gender* as a dichotomous variable.

*Marital status* at the time of the interview was divided into four categories: married/cohabiting, never married, divorced or separated, and widowed.

*Household’s social class* was based on the Swedish Socio-economic Index (SEI), which is based on occupation. Occupational groups are categorised on the basis of the length of education usually required for the type of occupation, typical trade union membership, position in the organisation (whether employed or self-employed), and size of the organisation (Andersson et al. 1981). In this study, the main occupation during the respondent’s working years was used. The variable was divided into five categories: intermediate/higher non-manual workers, lower non-manual workers, skilled manual workers, unskilled manual workers, and farmers or self-employed people. The social class position for the respondent’s household was assessed on the basis of information about the occupation of both the respondent and the respondent’s spouse. Both living and deceased spouses were considered. Thus, people who were widowed were categorised according to the deceased spouse’s social class (if it was the dominant class of the household). Moreover, divorced people were coded according to their previous spouse’s social class, however, less than half of the divorced people had a household social class that differed from the social class derived from their own occupation (*n* = 26).

When classifying people according to the household’s social class, we drew on Erikson’s (1984) argument that when spouses/cohabitants occupy different social classes, one of those social classes will have a greater influence on the household’s attitudes, behaviours, and consumption patterns. In other words, one dominant social class determines the household’s social class. Our classification was made by using a dominance scheme developed by Erikson (1984). For example, the employed dominate the unemployed, and among the employed, higher positions with higher qualifications dominate positions with lower qualifications.

### Statistical Analyses

Only people for whom there was enough information to create all variables were included in the analyses. Our final study sample consisted of 765 individuals. All outcomes under study – the disadvantages – were dichotomous. Analyses were carried out using Stata. Logistic regressions were used in several analyses. To facilitate interpretability and comparability across models, the results are presented as average marginal effects (AMEs) (Mood 2010). An AME can be interpreted as the average difference in the probability (0–1) of the outcome depending on the value of the independent variable. Moreover, we performed tetrachoric correlation analyses (Rho). The Rho correlation coefficient varies between −1 and 1, where −1 denotes a perfect negative association, and 1 denotes a perfect positive association. Sampling probability weights (Stata subcommand pweights) were used to adjust for the oversampling of the oldest age groups. The weights are the inverse of the probability that the observation is included because of the sampling design: those aged 85 years and older were given proportionally less weight in the analyses.

## Results

### Sample Characteristics

Table 1 shows the number and weighted percentage of people in the demographic and socio-economic groups. Approximately two thirds (63 %) of the respondents were women. The most common marital status was being married/cohabiting (45 %), and the second most common was being widowed (42 %). The largest social class category was intermediate/higher non-manual workers (36 %), followed by farmers and self-employed people (25 %). The people in the sample ranged in age from 76 to 101 years; their mean age was 83.

**Table 1:** Sample characteristics

	Freq.	Weighted %
Demographic and socio-economic characteristics		
Sex		
Men	340	37.5
Women	425	62.6
Marital status		
Married/cohabiting	307	45.1
Never married	31	4.4
Divorced/separated	56	8.9
Widowed	371	41.6
Household’s social class		
Intermediate/higher non-manual workers	249	35.9
Lower non-manual workers	99	12.4
Skilled manual workers	135	17.0
Unskilled manual workers	74	9.3
Farmers/self-employed	208	25.5
Age		
Range	76–101	
Mean, weighted	83	
Disadvantages		
Number of disadvantages		
0 disadvantages	220	32.6
1 disadvantage	266	35.5
2 or more (Coexisting disadvantages)	279	32.0
Single disadvantages		
Physical health problems	387	47.4
ADL limitations	224	22.2
Psychological health problems	76	9.6
Limited financial resources	80	11.8
Limited social resources	168	20.7
Single disadvantages among those reporting only one disadvantage (n = 266)		
Physical health problems	144	55.6
ADL limitations	49	13.5
Psychological health problems	8	3.4
Limited financial resources	16	8.0
Limited social resources	49	19.5
Combinations of disadvantages among those reporting coexisting disadvantages (n = 279)		
Physical + ADL	76	22.6
Physical + Psychological	15	7.6
Physical + Financial	17	8.1
Physical + Social	50	18.5
Physical + ADL + Psychological	18	4.1
Physical + ADL + Financial	15	6.3
Physical + ADL + Social	22	7.5
Physical + Psychological + Financial	3	1.7
Physical + Psychological + Social	4	2.3
Physical + Financial + Social	6	2.6
Physical + ADL + Psychological + Financial	6	3.0
Physical + ADL + Psychological + Social	6	1.3
Physical + ADL + Financial + Social	5	1.1
ADL + Psychological	7	2.7
ADL + Financial	3	1.0
ADL + Social	10	3.3
ADL + Psychological + Social	5	1.2
ADL + Financial + Social	2	0.4
Psychological + Social	2	1.1
Psychological + Financial + Social	2	1.1
Social + Financial	5	2.6

Table 1 also shows the number and weighted percentage of people who reported coexisting disadvantages and the various single disadvantages. The frequencies for no disadvantages, one disadvantage and coexisting disadvantages were similar in size – around 30 %. Reporting one disadvantage was most common (35.5 %), but the difference was limited to a few percentage points. The most common disadvantage was physical health problems (47 %) followed by ADL limitations (22 %) and limited social resources (21 %). Among those who reported *one* disadvantage, the majority (56 %) experienced physical health problems, 20 % experienced limited social resources and 14 % reported ADL limitations. Psychological health problems and limited financial resources were reported less frequently.

As shown in the table, around one third of people in the sample reported two or more coexisting disadvantages, which comprises various combinations of disadvantages. In our sample, 21 unique combinations of disadvantages emerged. The combinations mostly consisted of two or three simultaneous disadvantages. Out of those reporting coexisting disadvantages, 86.5 % experienced a combination that included physical health problems. The most common combination, physical health problems and ADL limitations, was reported by 22.6 % of those who experienced coexisting disadvantages. The second most common combination was physical health problems and limited social resources (18.5 %), followed by physical health problems and limited financial resources (8.1 %), physical health problems and psychological health problems (7.6 %), and physical health problems, ADL limitations and limited social resources (7.5 %).

### Number of Disadvantages in Different Demographic and Socio-Economic Groups

Table 2 shows differences in the probability of reporting no disadvantages, one disadvantage (regardless of type), and of reporting coexisting disadvantages (regardless of combination) between different demographic and socio-economic groups. The results are derived from multinomial regression analyses. First, we analysed bivariate models and as a second step, we analysed fully adjusted models where all the covariates were included.

**Table 2:** Associations between demographic and socio-economic indicators and number of disadvantages. Average marginal effects (AMEs) derived from bivariate and multivariate multinomial regression analyses

	No disadvantages				1 Disadvantage				Coexisting disadvantages (2+)			
Demographic and socio-economic indicators	Bivariate model		Full model		Bivariate model		Full model		Bivariate model		Full model	
	AME	P-value	AME	*P*-value	AME	P-value	AME	P-value	AME	P-value	AME	P-value
*Age*												
Continuous	**-0.018**	**0.000**	**-0.015**	**0.000**	0.002	0.534	0.002	0.522	**0.016**	**0.000**	**0.013**	**0.000**
*Sex*												
Men	(ref)		(ref)		(ref)		(ref)		(ref)		(ref)	
Women	**-0.106**	**0.004**	-0.033	0.397	0.018	0.654	0.010	0.828	**0.088**	**0.021**	0.024	0.549
*Marital status*												
Married/cohabiting	(ref)		(ref)		(ref)		(ref)		(ref)		(ref)	
Never married	**-0.192**	**0.034**	-0.141	0.117	-0.027	0.778	-0.040	0.683	**0.219**	**0.028**	0.181	0.054
Divorced/separated	**-0.338**	**0.000**	**-0.301**	**0.000**	0.092	0.218	0.073	0.343	**0.246**	**0.001**	**0.228**	**0.003**
Widowed	**-0.199**	**0.000**	**-0.121**	**0.009**	0.024	0.560	0.011	0.819	**0.175**	**0.000**	**0.110**	**0.011**
*Household’s social class*												
Intermediate/higher non-manual workers	(ref)		(ref)		(ref)		(ref)		(ref)		(ref)	
Lower non-manual workers	-0.089	0.153	-0.053	0.371	0.040	0.543	0.036	0.586	0.050	0.413	0.017	0.765
Skilled manual workers	**-0.121**	**0.030**	-0.079	0.158	0.007	0.902	-0.000	0.996	**0.114**	**0.040**	0.079	0.161
Unskilled manual workers	**-0.272**	**0.000**	**-0.217**	**0.000**	0.053	0.471	0.052	0.490	**0.219**	**0.003**	**0.166**	**0.022**
Farmers/self-employed	-0.052	0.311	0.008	0.870	-0.027	0.580	-0.038	0.447	0.079	0.096	0.030	0.525
n	765											
Pseudo R2	0.063 (Full model)											

*P*-values <0.05 are indicated in bold

The results from all analyses showed that several groups were less likely than other groups to report no disadvantages and the same groups were more likely to report coexisting disadvantages. In the fully adjusted model, these groups were older people, divorced/separated people, widowed people and unskilled manual workers. There were no statistically significant differences between the groups in the probability of reporting one disadvantage.

In the full model, the probability of reporting no disadvantages decreased by 1.5 percentage points for every one-year increase in age (AME −0.015). On the other hand, the probability of reporting coexisting disadvantages indicates that for every one-year increase in age, the probability of reporting coexisting disadvantages increased by an average of 1.3 percentage points (AME 0.013).

In the bivariate analyses, the probability of reporting no disadvantages was, on average, 10.6 percentage points lower for women than for men (AME −0.106). The estimate for coexisting disadvantages (AME 0.088) suggests that women had, on average, 8.8 percentage points higher probability of experiencing coexisting disadvantages than men. When all the demographic and socio-economic variables were simultaneously included in the full models, the associations between sex and coexisting disadvantages attenuated and fell below statistical significance. The change was mainly attributable to the inclusion of marital status (not shown). Those who were never married, divorced or widowed were substantially less likely to report no disadvantages, and more likely to report coexisting disadvantages than those who were married/cohabiting. Moreover, a higher proportion of women than men were found in marital status categories other than ‘married/cohabiting’. Fifty-five percent of the women were widowed and 10 % were divorced, whereas 20 % of the men were widowed and 6 % were divorced (not shown). Thus, the high proportion of women in the divorced and the widowed groups may explain their increased likelihood of being exposed to coexisting disadvantages.

Differences between social classes were mainly found between manual workers and the reference group, intermediate/higher non-manual workers. The probability to report no disadvantages was higher for unskilled manual workers than those in the reference group. Moreover, unskilled manual workers had a higher probability than the reference group to report coexisting disadvantages. The same pattern was found for skilled manual workers in the bivariate analyses. The full models showed no statistically significant differences between skilled manual workers and intermediate/higher non-manual workers. Still, the analyses indicated that skilled manual workers tended to be less likely to experience no disadvantages and more likely to experience two or more disadvantages. Age, sex, and marital status all contributed to the reduction in the estimates.

### Specific Disadvantages in Different Demographic and Socio-Economic Groups

As previously mentioned, research suggests that certain demographic and socio-economic groups tend to be associated with disadvantage expressed in general terms (such as number of disadvantages) but need not necessarily be disadvantaged in all life domains (Barnes et al. 2006). To test whether this was also the case in our study sample, we analysed the probability that the demographic and socio-economic groups would experience each of the four individual disadvantages. The results are shown in Table 3.

**Table 3:** Associations between demographic and socio-economic indicators and single disadvantages. Average marginal effects (AMEs) derived from bivariate and multivariate logistic regression analysis

Demographic and socio-economic indicators	Physical health problems				ADL limitations				Psychological health problems				Limited financial resources				Limited social resources			
	Bivariate		Full model		Bivariate		Full model		Bivariate		Full model		Bivariate		Full model					
	AME	P-value	AME	P-value	AME	P-value	AME	P-value	AME	P-value	AME	P-value	AME	P-value	AME	P-value	AME	P-value	AME	P-value
Age																				
Continuous	**0.012**	**0.001**	**0.007**	**0.046**	**0.025**	**0.000**	**0.022**	**0.000**	0.001	0.550	0.001	0.739	-0.000	0.852	-0.001	0.706	**0.006**	**0.019**	**0.006**	**0.035**
Sex																				
Men	(ref)		(ref)		(ref)		(ref)		(ref)		(ref)		(ref)		(ref)		(ref)		(ref)	
Women	0.028	0.496	-0.051	0.248	**0.098**	**0.004**	0.051	0.139	**0.054**	**0.049**	*0.053*	*0.053*	**0.069**	**0.021**	0.040	0.159	0.050	0.144	0.013	0.727
Marital status																				
Married/cohabiting	(ref)		(ref)		(ref)		(ref)		(ref)		(ref)		(ref)		(ref)		(ref)		(ref)	
Never married	0.054	0.594	0.028	0.782	0.111	0.191	0.049	0.493	0.047	0.493	0.041	0.525	0.101	0.186	0.083	0.170	**0.307**	**0.002**	**0.308**	**0.002**
Divorced/separated	**0.208**	**0.005**	**0.218**	**0.003**	0.090	0.147	0.083	0.205	-0.014	0.733	-0.025	0.547	**0.239**	**0.000**	**0.198**	**0.001**	**0.181**	**0.007**	**0.184**	**0.007**
Widowed	**0.184**	**0.000**	**0.172**	**0.000**	**0.164**	**0.000**	0.051	0.154	0.022	0.392	-0.001	0.983	**0.064**	**0.014**	*0.049*	*0.069*	**0.108**	**0.001**	**0.091**	**0.018**
Household’s social class																				
Intermediate/higher non-manual workers	(ref)		(ref)		(ref)		(ref)		(ref)		(ref)		(ref)		(ref)		(ref)		(ref)	
Lower non-manual workers	**0.150**	**0.023**	**0.133**	**0.038**	**0.122**	**0.025**	*0.087*	*0.086*	0.061	0.168	0.054	0.212	-0.022	0.535	-0.030	0.396	-0.073	0.156	**-0.103**	**0.039**
Skilled manual workers	0.041	0.489	0.017	0.776	**0.153**	**0.002**	**0.109**	**0.024**	0.065	0.100	0.057	0.145	0.039	0.315	0.030	0.460	0.019	0.712	-0.005	0.929
Unskilled manual workers	*0.127*	*0.088*	0.092	0.222	**0.148**	**0.018**	*0.105*	*0.064*	-0.022	0.531	-0.027	0.430	**0.215**	**0.001**	**0.173**	**0.005**	0.041	0.534	-0.010	0.878
Farmers/self-employed	*0.094*	*0.073*	0.060	0.248	**0.147**	**0.000**	**0.081**	**0.041**	0.001	0.979	-0.005	0.863	0.006	0.859	-0.002	0.957	**-0.101**	**0.010**	**-0.129**	**0.001**
*n*	765																			
Pseudo R2			0.038				0.138				0.029				0.090				0.062	

*P*-values <0.05 are indicated in bold

In sum, the results show that different demographic and socio-economic groups tended to be associated with different kinds of disadvantages. Older age was associated with an increased probability of experiencing physical health problems, ADL limitations, and of experiencing limited social resources. Being female increased the probability of experiencing psychological health problems. Being female was associated with ADL limitations and limited financial resources in the bivariate models but not in the full models. The associations weakened mainly because of the inclusion of marital status.

People who had never been married were more likely to report limited social resources than married/cohabiting people. Divorced and widowed people had an increased probability of reporting physical health problems, limited financial resources, and limited social resources. In the bivariate analysis, widowed people were more likely than married people to report ADL limitations. In the full model, the association between widowhood and ADL limitations weakened and fell below statistical significance.

Lower non-manual workers were *more* likely to report physical health problems and ADL limitations, and were *less* likely to report limited social resources than the reference group, intermediate/higher non-manual workers. Skilled manual workers had an increased probability of reporting ADL limitations, and unskilled manual workers had an increased probability of reporting ADL limitations and limited financial resources. Farmers and self-employed people were *more* likely to report ADL limitations and *less* likely to report limited social resources than the reference group.

### Combinations of Disadvantages in Different Demographic and Socio-Economic Groups

Since the vast majority of those afflicted by coexisting disadvantages reported physical health problems as one of the disadvantages, we limited our further analyses to combinations of disadvantages that included physical health problems. As a first step, we explored whether there were any correlations between physical health problems and the other disadvantages under study. The results are shown in Table 4.

**Table 4:** Bivariate tetrachoric correlations between physical health problems and other disadvantages

		Rho	CI (95 %)
Physical health problems	ADL limitations	**0.34**	**0.22–0.47**
Physical health problems	Psychological health problems	**0.28**	**0.11–0.38**
Physical health problems	Limited financial resources	**0.22**	**0.06–0.38**
Physical health problems	Limited social resources	0.07	-0.07 – 0.21
	n	765	

*P*-values <0.05 are indicated in bold

In sum, in the older general older population, the associations between physical health problems and other disadvantages were moderate to low. The strongest association was found between physical health problems and ADL limitations. Moderate associations were found between physical health problems and psychological health problems, and physical health problems and limited financial resources. The results showed no association between physical health problems and limited social resources.

As a second step, we analysed demographic and socio-economic differences in the probabilities of reporting different combinations of disadvantages including physical health problems. Hence, we created four pairwise combinations of disadvantages, all of which included physical health problems.

The analysed combinations were: 1) physical health problems and ADL limitations (weighted percent of those who experienced coexisting disadvantages = 14.6, *n* = 148), 2) physical health problems and psychological health problems (weighted percent = 6.4, *n* = 52), 3) physical health problems and limited financial resources (weighted percent = 7.3, n = 52), and 4) physical health problems and limited social resources (weighted percent =10.6, *n* = 93). People who reported three simultaneous disadvantages were included in two different combinations. For example, if someone reported physical health problems, psychological health problems, and limited financial resources, this person was included in combinations 2 and 3.

The probability that the people in the different demographic and socio-economic groups would experience the four combinations of disadvantages is shown in Table 5. In general, different socio-demographic groups tended to be associated with different combinations of disadvantages.

**Table 5:** Associations between socio-demographic indicators and combinations of disadvantages. Average marginal effects (AMEs) derived from bivariate and multivariate regression analyses

Demographic and socio-economic indicators	Physical health problems + ADL limitations				Physical health problems + Psychological health problems				Physical health problems + Limited financial resources				Physical health problems + Limited social resources			
	Bivariate		Full model		Bivariate		Full model		Bivariate		Full model		Bivariate		Full model	
	AME	P-value	AME	P-value	AME	P-value	AME	P-value	AME	P-value	AME	P-value	AME	P-value	AME	P-value
Age																
Continuous	**0.018**	**0.000**	**0.015**	**0.000**	-0.000	0.929	-0.001	0.518	0.001	0.555	0.000	0.808	**0.005**	**0.010**	0.004	0.064
Sex																
Men	(ref)		(ref)		(ref)		(ref)		(ref)		(ref)		(ref)		(ref)	
Women	**0.072**	**0.015**	0.017	0.526	0.025	0.251	0.010	0.624	0.047	0.056	0.024	0.337	0.009	0.728	-0.029	0.264
Marital status																
Married/cohabiting	(ref)		(ref)		(ref)		(ref)		(ref)		(ref)		(ref)		(ref)	
Never married	0.116	0.124	0.078	0.212	0.001	0.980	0.002	0.954	0.007	0.870	-0.002	0.961	0.137	0.070	0.137	0.069
Divorced/separated	0.090	0.087	0.100	0.092	0.027	0.471	0.027	0.477	**0.166**	**0.004**	**0.146**	**0.008**	0.085	0.083	0.098	0.064
Widowed	**0.156**	**0.000**	**0.087**	**0.002**	0.040	0.061	**0.042**	**0.047**	**0.052**	**0.013**	0.040	0.075	**0.092**	**0.000**	**0.095**	**0.001**
Household’s social class																
Intermediate/higher non-manual workers	(ref)		(ref)		(ref)		(ref)		(ref)		(ref)		(ref)		(ref)	
Lower non-manual workers	0.087	0.057	0.064	0.141	0.025	0.503	0.022	0.537	0.021	0.537	0.017	0.596	-0.019	0.637	-0.036	0.370
Skilled manual workers	**0.100**	**0.011**	0.069	0.073	0.020	0.536	0.015	0.635	0.029	0.333	0.021	0.484	-0.012	0.747	-0.027	0.461
Unskilled manual workers	**0.107**	**0.049**	0.076	0.124	-0.027	0.339	-0.031	0.260	**0.108**	**0.041**	0.084	0.088	0.016	0.752	-0.010	0.842
Farmers/self-employed	**0.122**	**0.001**	**0.073**	**0.034**	-0.013	0.577	-0.017	0.500	0.024	0.361	0.017	0.538	-0.041	0.171	**-0.060**	**0.048**
*n*	765															
Pseudo R2			0.143				0.025				0.072				0.054	

*P*-v alues <0.05 are indicated in bold

Older age was associated with an increased probability of experiencing the combinations physical health problems and ADL limitations, and physical health problems together with limited social resources. In the bivariate models, being female was associated with a higher probability of experiencing the combination physical health problems and ADL limitations, and tended to be associated with the combination physical health problems and limited financial resources. In the full model, the estimates attenuated and fell below statistical significance. This was mainly attributable to the inclusion of marital status in the analyses.

Those who had never been married tended to be more likely to experience a combination of physical health problems and limited social resources than those who were married/cohabiting. Divorced people were more likely than married/cohabiting people to experience a combination of physical health problems and limited financial resources and also tended to be more likely to experience a combination of physical health problems and ADL limitations, and a combination of physical health problems and limited social resources. Widowed people were more likely than married/cohabiting people to report all the analysed combinations of disadvantages.

Unskilled manual workers were more likely to experience a combination of physical health problems and a limited financial resources than intermediate/higher non-manual workers, and in the bivariate analysis, were also more likely to report a combination of physical health problems and ADL limitations. Finally, farmers and self-employed people were more likely than intermediate/higher non-manual workers to report a combination of physical health problems and ADL limitations, and were less likely than the reference group to report a combination of physical health problems and limited social resources.

## Discussion

In this study we explored demographic and socio-economic inequality in the probability that older adults in Sweden would experience coexisting disadvantages. To do this, we compared analyses of coexisting disadvantages, measured as two or more simultaneous disadvantages, with analyses of single indicators and specific combinations of disadvantages. Indicators of physical health problems, ADL limitations, psychological health problems, limited financial resources, and limited social resources were used.

Previous studies have predominantly found that in older people, advanced old age is associated with a higher probability of experiencing coexisting disadvantages (Barnes et al. 2006; Halleröd 2009; Heap et al. 2013; Tsakloglou and Papadopoulos 2002), a pattern also seen in our results: higher age was associated with an increased probability of reporting two or more coexisting disadvantages. Some previous studies, however, have found the opposite (Bask 2015), possibly because they included a set of indicators which mainly measured psychosocial distress.

Studies of gender differences in the probability of experiencing coexisting disadvantages have shown mixed results (Bask 2010; Ferrarini et al. 2010; Halleröd and Larsson 2008; Heap et al. 2013; Korpi et al. 2007). In this study, we found differences between women and men in several bivariate analyses. However, these were attributable to differences in marital status; that is, women were more often divorced or widowed than men. Still, as previous studies have generally included marital status or living arrangements (e.g. living alone or cohabiting) in their analyses, the explanation of the diverging gender patterns probably does not lie in differences regarding control for such variables. Thus, further explanations of the differing results of gender differences need to be sought. One possibility is that the studies used different measures of disadvantage. Although most of the studies covered similar life domains, the domains and indicators included in the studies varied somewhat, and patterns of gender inequality may be sensitive to these variations.

Considerable social class differences in the probability of experiencing coexisting disadvantages have been found in previous studies of people of working age and young old age (Fritzell and Lundberg 2000; Halleröd and Larsson 2008; Korpi et al. 2007). Our results show that social class also influences the likelihood that older people will experience coexisting disadvantages. Another consistent pattern among people of working age and young old age is that divorced people and people who live in single-headed households are more likely to experience coexisting disadvantages than married or cohabiting people (Bask 2010; Halleröd and Larsson 2008; Korpi et al. 2007; Muffels and Fouarge 2004; Whelan and Maître 2007). Since the majority of those in our sample who were not married/cohabiting lived alone (98.3 %), our results are comparable with those of previous studies. Our findings thus suggest that the pattern found in younger people persists into old age, and that marital status is a significant stratifying characteristic in older people. In addition, marital status could explain gender differences in the likelihood of experiencing coexisting disadvantages. Women were more often divorced or separated than men which contributed to the increased probability of experiencing coexisting disadvantages among women.

One explanation for the stratifying effect of marital status can be that marriage itself is beneficial for health and other aspects of living conditions. For example, the marital resource model suggests that differences in health between marital status groups can be ascribed to the higher financial resources and social support that married people hold, as well as regulations of health behaviours that come from marriage (Williams and Umberson 2004). However, research has shown that there is also a selection into different categories of marital status. For example, those who have health problems or experience other kinds of disadvantages are less likely to enter, and stay in, a stable relationship (e.g. Halleröd and Bask 2008).

It is noteworthy that the differences between the demographic and socio-economic groups were only found for those who reported coexisting disadvantages and not for those who reported only one disadvantage. This is in line with the findings of a study that explored different measures of poverty, where it was found that the more dimensions of poverty people were exposed to, the more their demographic and socio-economic characteristics differed from the characteristics of those who were not poor (Bradshaw and Finch 2003). Demographic and social factors may thus become more important as disadvantages compound.

### Different Groups – Different Disadvantages

Studies of coexisting disadvantages that focus on people of working age have found that limited financial resources are often associated with other disadvantages (Fritzell and Lundberg 2000; Halleröd and Bask 2008; Korpi et al. 2007). Among older people, physical health problems seem to play a similarly central role (Heap and Fors 2015; Heap et al. 2013; Whelan and Maître 2008), a finding confirmed in this study. Our results showed that physical health problems were reported by a majority of those who experienced coexisting disadvantages.

The analyses of separate domains of disadvantage showed that although certain demographic and socio-economic groups were associated with coexisting disadvantages, no such groups were disadvantaged in all of the life domains we analysed. This pattern has previously been noted by Barnes et al. (2006). The analyses of specific combinations of disadvantages showed that the groups associated with coexisting disadvantages were exposed to different combinations of disadvantages. We found that unskilled manual workers and divorced people were more likely to experience a combination of physical health problems and limited financial resources than people in the reference categories. The combination of physical health problems and limited social resources was associated with older age, with never having married, with being widowed, and with being divorced. The groups that had these characteristics were all more likely to report the single indicators that were included in the combinations of disadvantages. Accordingly, no new groups emerged that were more likely to be exposed to a combination that was not observable in the results of the analyses of single domains.

### Limitations and Strengths

The results of this study may have been different if we had included other kinds of disadvantages. However, the disadvantages analysed in this study have often been used in previous empirical studies and are considered central domains of disadvantage in the Scandinavian tradition of welfare research (Allardt 1993; Johansson 1970) and in related research fields (e.g. Stiglitz et al. 2009). Moreover, as discussed previously, different operationalisations may also affect results, and hence patterns of inequality. Furthermore, our small sample size prevented us from analysing all the combinations of disadvantages that were found. It would, for example, have been of interest to include the combinations of three disadvantages in the analyses, since it is possible that the higher the number of disadvantages that people experience, the more their social background differs from people who do not experience any disadvantages (Bradshaw and Finch 2003) – a pattern which was partly confirmed in our study.

One problem in surveys of the older population using self-reported measures of physical and psychological health is that people with cognitive impairment may have difficulties responding to such questions. Proxy interviews and mixed interviews (where both the respondent and a proxy are present) have been used in the SWEOLD survey to limit this problem. Thus, proxy interviews facilitate participation among people who are cognitive impaired or may not be able to participate because of other health problems. It has been shown that excluding proxy interviews from the SWEOLD study leads to an underestimation of health problems in the older Swedish population (Kelfve 2015).

The strengths of this study included the use of a nationally representative sample and the high response rate. Moreover, the sample included people who lived in an institution, a group that is often left out of surveys of older people.

### Concluding Remarks

Social class patterns found among people of working age and young old age were also present in our sample of people aged 76 and older. Our results also suggest that marital status is important to the likelihood that older people will experience coexisting disadvantages Divorced people seemed to be a particularly disadvantaged group, and unlike other groups, were associated with more than one specific combination of disadvantages. Moreover, marital status could explain gender differences in patterns of coexisting disadvantages. That is, the higher prevalence of divorce and widowhood among women than men explained women’s disadvantaged position relative to men.

Furthermore, our results show that coexisting disadvantages are more unequally distributed in the older population than single disadvantages. These results resonate with cumulative inequality theory in suggesting that disadvantages tend to accumulate in certain groups over the life course.

People who experience coexisting disadvantages may need health care, social care, or other types of support. Since the demographic and socio-economic groups that were associated with coexisting disadvantages tended to experience different combinations of disadvantages, policy interventions targeted at vulnerable groups should be designed with regard to different profiles of disadvantage. Different combinations of disadvantages imply different kinds of hardship. They may also imply the need for different kinds of help and support.

Physical health problems play a central role in the coexistence of disadvantages among older people. Our analyses suggest that the significance of this disadvantage lies in its high frequency. Because of the moderate associations between physical health problems and the other disadvantages analysed in this study, it seems less plausible that physical health problems are included in a series of causal associations between disadvantages, at least not in the general older population. It is, however, possible that this is the case in certain sub-groups of the population. Another possibility is that social position per se, such as being divorced, entails an increased risk of several disadvantages that are relatively independent of each other. In this study, we focused on identifying demographic and socio-economic groups that are vulnerable to coexisting disadvantages. Teasing out causal mechanisms is an important task for future studies.

## Footnotes

1 To minimise the amount of missing cases due to internal non-response in one or more of the items included in the variable, people who had missing values on certain (but not all) items and had sufficient problems to be classified as being in poor psychological health were included in the variable of psychological health problems.

2 The previously mentioned procedure to minimise the number of missing cases was also carried out for the index of physical health problems.
